# Tissue Effects of the Mitochondrial Division Inhibitor Mdivi-1 on the Substantia Nigra in a Laboratory Model of Dopaminergic System Damage

**DOI:** 10.3390/ijms27115003

**Published:** 2026-06-01

**Authors:** Anna V. Egorova, Dmitry N. Voronkov, Maria S. Ryabova, Alla V. Stavrovskaya, Artem S. Olshansky, Anastasia K. Pavlova, Tatiana I. Baranich, Dmitry A. Kharlamov, Vladimir S. Sukhorukov

**Affiliations:** 1 Laboratory of Neuromorphology, Russian Center of Neurology and Neurosciences, 125367 Moscow, Russia; voronkov@neurology.ru (D.N.V.); ryabovamarias@gmail.com (M.S.R.); alla_stav@mail.ru (A.V.S.); as0769@yandex.ru (A.S.O.); pav_nastasya@mail.ru (A.K.P.); baranich_tatyana@mail.ru (T.I.B.); vsukhorukov@gmail.com (V.S.S.); 2 Department of Morphology, Pirogov Russian National Research Medical University, 117513 Moscow, Russia; 3 Department of Anatomy and Histology, I. M. Sechenov First Moscow State Medical University (Sechenov University), 125009 Moscow, Russia; 4 V. F. Voyno-Yasenetsky Scientific and Practical Center of Specialized Medical Care for Children, 119620 Moscow, Russia; dmitoch@gmail.com

**Keywords:** Parkinson’s disease, 6-hydroxydopamine, substantia nigra, microglia, mitochondrial dynamics, Mdivi-1

## Abstract

The use of substances that modulate mitochondrial dynamics in cells of nervous tissue represents a new direction in targeted therapy for neurodegeneration. The aim of this study was to evaluate the effect of mitochondrial division inhibitor-1 (Mdivi-1) on neurons and microgliocytes of the substantia nigra under conditions of partial damage to the dopaminergic system. This study was conducted using the 6-hydroxydopamine model of parkinsonism in rats; a separate experimental group of animals received Mdivi-1 intraperitoneally at a dose of 20 mg/kg for 5 days. The intensity of immunofluorescence staining for Tomm20, MTCO1, pDrp1, and Mfn2 was evaluated in neurons of the substantia nigra, and microglial activation was morphologically assessed. It was found that unilateral administration of 6-OHDA led to pro-inflammatory changes in microglia and changes in mitochondrial markers of neurons on the side of the substantia nigra contralateral to the toxin injection. Mdivi-1 did not affect the damage and mitochondrial proteins of neurons in pars compacta of the substantia nigra; however, it changed mitochondrial markers in nervous cells of the pars reticulata. The use of Mdivi-1 to address abnormal processes in neurodegeneration requires additional studies that include a differential assessment of its effects on various cell types.

## 1. Introduction

Parkinson’s disease (PD) is a progressive neurodegenerative disorder associated with the loss of dopaminergic neurons in the substantia nigra (SN) of the midbrain. Significant progress has been made recently in studying PD patterns; however, its molecular basis remains understudied. The key pathogenetic mechanisms leading to progressive neuronal loss are neuroinflammation, mitochondrial dysfunction, oxidative stress, increased blood–brain barrier permeability, impaired glio-neuronal interactions, and disruption of multiple signaling pathways [1].

In the context of studying PD pathogenesis, mitochondria have become a subject of heightened research interest in recent years. Changes in mitochondrial dynamics and the associated possible manifestations of mitochondrial dysfunction in nervous cells have been well described in neurodegenerative processes; however, the findings obtained are highly contradictory [2]. Most authors modeling parkinsonism experimentally report a tendency toward increased mitochondrial fission in neurons of the substantia nigra, accompanied by impaired mitophagy and mitochondrial dysfunction [3]. However, mitochondrial fission, mediated by the dynamin family GTPase Drp1, can occur either as proliferation or fragmentation depending on the initial functional state of the organelles and may be either compensatory or abnormal [4]. It is traditionally believed that fusion proteins (Mfn1, Mfn2, OPA1) improve the bioenergetic state of mitochondria and influence calcium homeostasis and reactive oxygen species production [5]. However, excessive stimulation of mitochondrial fusion leads to the appearance of elongated defective organelles and reduced efficiency of mitophagy, underscoring the need to maintain a balance in mitochondrial dynamics. In this regard, the search for new therapeutic approaches that allow the administration of substances modulating mitochondrial dynamics into the comprehensive treatment of the disease is of particular interest [6].

In the context of the above, the mitochondrial division inhibitor Mdivi-1, which is capable of crossing the blood–brain barrier, has attracted the attention of researchers. Many recent studies demonstrate that this compound possesses neuroprotective properties, such as anti-apoptotic effects, optimization of calcium homeostasis, and reduction in reactive oxygen species production [7,8]. There are reports that Mdivi-1 can suppress neuroinflammation by reducing the production of pro-inflammatory factors by activated microglia [9].

Despite numerous data on the positive effects of Mdivi-1, there have recently been works that cast doubt on the initial opinion about the selectivity of its action and demonstrate the direct binding and inhibitory effect of Mdivi-1 on mitochondrial complex I [10,11]. Specifically, the work by N. Marx et al. demonstrated that mitochondrial division inhibitor disrupts the assembly of complex I of mitochondria and respiratory supercomplexes required for efficient electron transport, thereby reducing ATP production and adversely affecting calcium homeostasis. Given that inhibition of the electron transport chain, particularly complex I, is closely associated with the onset of neurodegeneration and that data on the metabolism and pharmacokinetics of Mdivi-1 in the literature are insufficient, numerous issues regarding the mechanism of action of mitochondrial division inhibitor (both dependent on and independent of its effects on Drp-1) remain to be resolved.

The wide range of substantia nigra functions underlies the structural and neurochemical heterogeneity of its neurons. Functionally and morphologically, this brain region is divided into the pars compacta (SNpc), which contains dopaminergic neurons that send projections to the striatum, and the pars reticulata (SNpr), which contains nervous cells that produce gamma-aminobutyric acid (GABA) and serves as the output from the basal ganglia system to the thalamic nuclei.

Dopaminergic and GABAergic neurons of the substantia nigra mutually modulate their activity both through direct interactions and through connections within the basal ganglia system [12,13,14]. At the same time, few studies have assessed changes in the pars reticulata of the substantia nigra in experimental models of PD. Various experimental models have shown that damage to dopaminergic neurons of the substantia nigra leads to disturbances in the activity of nerve cells in the pars reticulata: alterations in spike frequency and regularity, increased burst activity, and synchronization [15]; moreover, the transition to an abnormal state is observed before the manifestation of motor symptoms. Experiments have found increased GABA and glutamate levels, as well as an increased density of GABAergic synapses on neurons of the SNpr on the side of 6-hydroxydopamine (6-OHDA) injection [16]. Morphological studies concerning neurons of the pars reticulata of the substantia nigra in patients with PD are few; for example, early studies reported a decreased density of non-dopaminergic neurons, whereas other authors have indicated an absence of their degeneration [17]. According to classical concepts, the reduction in dopamine levels resulting from the death of neurons in the pars compacta in PD leads to increased activity of GABAergic neurons in the SNpr, which exert an inhibitory influence on thalamic neuron activity. In this context, inhibition of pars reticulata neurons in PD models alleviates motor deficits in animals [18,19].

In experiments with neurotoxins, the half of the substantia nigra contralateral to the toxin injection is considered as a control. At the same time, unilateral toxin injection can also be viewed as a reproduction of the asymmetric PD, which is quite often observed in the early stages of the disease in humans [20]. Given the anatomical and functional interconnections of the basal ganglia, it is conceivable either that compensation of neurotransmitter imbalance occurs following damage to dopaminergic neurons of the substantia nigra or, conversely, that cells on the contralateral side become involved in the spread of the abnormal process.

Currently, the most popular research tool is modeling Parkinson’s disease using toxins that cause selective death of dopaminergic neurons. One frequently used substance is 6-OHDA, which is structurally similar to dopamine and norepinephrine. After intracerebral administration, the neurotoxin enters cells, undergoes metabolic degradation, and produces hydrogen peroxide, superoxide anion, and hydroxyl radicals, ultimately leading to oxidative stress and mitochondrial dysfunction [21]. Along with the death of dopaminergic cells, pronounced gliosis is typical for the hydroxydopamine model.

Initially, microglial activation may act as a neuroprotective mechanism by clearing dead cells and their debris, removing excess neurotoxins, and stimulating repair processes. Enhanced and prolonged microglial activation leads to cytotoxic effects, accelerating neuronal damage. Thus, microglial cells play a dual role in brain tissue injury, exerting both damaging and restorative effects on neurons, which indicates the need for a detailed study of this issue using diverse experimental approaches [22].

Inflammatory stimuli alter microglial morphology. In the classical view, upon activation, microgliocytes undergo several successive stages, which may include: increased process arborization, cell body hypertrophy, cell polarization and elongation with a reduction in the number of processes, and the formation of rod-shaped and amoeboid forms. Unlike the ramified state, amoeboid microglia exhibit high levels of phagocytosis, migratory capacity, and secretion of pro-inflammatory factors. However, it appears that, under different conditions, the morphological and functional changes in microglia vary considerably depending on the stimulus nature [23]. The transition of microglia to an activated state is accompanied by increased mitochondrial fission.

Despite its long history of use, the 6-OHDA model of parkinsonism has not provided answers regarding the nuances of changes in mitochondrial dynamics that take into account the cellular heterogeneity of the substantia nigra. Few studies have addressed the mechanisms of Mdivi-1 action on microglial cells, and some authors have not confirmed the neuroprotective effects of this compound in toxic models of neurodegenerative conditions, thus warranting further research in the field [24].

This study was aimed to evaluate the effect of Mdivi-1 on neurons and microgliocytes in the pars compacta and pars reticulata of the substantia nigra under conditions of partial damage to the dopaminergic system in an experimental setting.

## 2. Results

### 2.1. Assessment of Neurodegeneration in the Substantia Nigra

**Figure 1:**
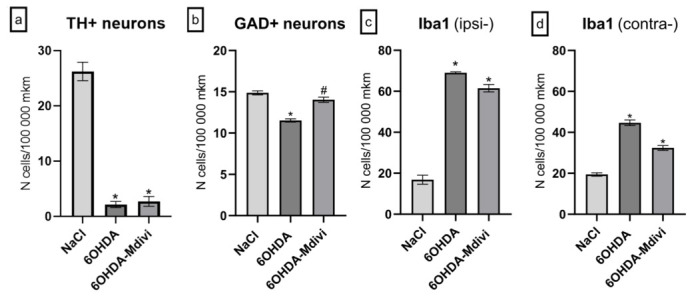
Quantitative assessment of changes in cell populations in the substantia nigra. (**a**) Change in dopaminergic (TH-positive) neuron density in the SNpc on the lesioned side; (**b**) change in the number of GABAergic (GAD-positive) neurons on the lesioned side; (**c**) change in Iba-positive cell density on the lesioned side (ipsi-); (**d**) contralateral side (contra-). ANOVA with post hoc Tukey test (M ± SEM). “*” *p* < 0.05 vs. control; “#” *p* < 0.05 vs. 6-OHDA. NaCl—group receiving isotonic NaCl injections (*n* = 5); 6-OHDA—animals receiving hydroxydopamine (*n* = 5); 6-OHDA-Mdivi—animals receiving the toxin and Mdivi-1 (*n* = 5).

**Figure 2:**
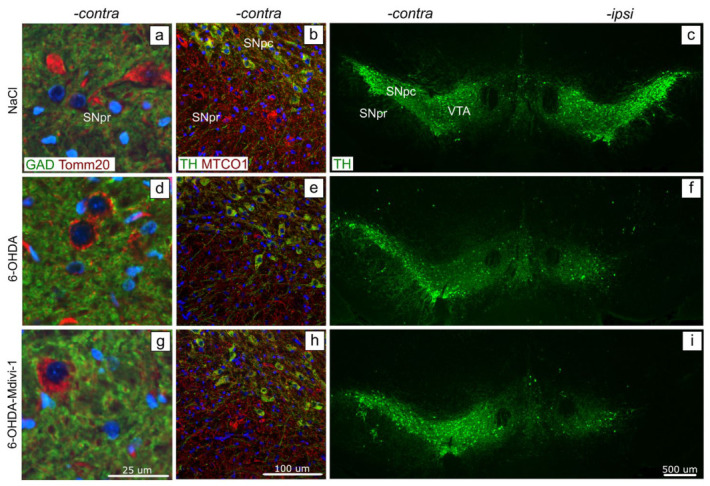
Immunofluorescence staining of substantia nigra neurons. (**a**,**d**,**g**) Detection of glutamate decarboxylase (GAD, green), Tomm20 (red) and DAPI (blue) ×20 objective; (**b**,**e**,**h**) detection of tyrosine hydroxylase (TH, green) and MTCO1 (red), ×20 objective; (**c**,**f**,**i**) assessment of dopaminergic neuron damage (TH, green). SNpr—substantia nigra pars reticulata; SNpc—substantia nigra pars compacta; VTA—ventral tegmental area. For group designations, see Figure 1.

Administration of 6-OHDA to the experimental group was accompanied by massive death of TH-positive neurons (Figure 1a and Figure 2) on the injection side of the toxin, which was confirmed by measuring the density of neurons in the SNpc, which was significantly lower (*p* < 0.0001) compared to the injection side in the control group. The intensity of immunofluorescence staining for TH on the toxin injection side was significantly reduced (*p* < 0.0001) compared to the control (48%) and the contralateral side (52%). The relatively high level of staining on the lesioned side is associated with the presence of cellular detritus and non-specific autofluorescence and does not fully correspond to the number of preserved neurons.

Although 6-OHDA exerts a selective damaging effect on dopamine neurons, the experiment also revealed its negative impact on GABAergic cells (GAD-positive neurons): toxin injection was found to reduce their density in the SNpr on the lesioned side compared to the control (*p* = 0.0054) (Figure 1 and Figure 2).

Treatment by Mdivi-1 did not significantly affect the indicators of dopaminergic neuron degeneration: the density and fluorescence intensity of TH-positive neurons remained significantly lower than control values. In animals of the same group, the number of GAD-positive cells on the toxin injection side tended to control levels (Figure 1 and Figure 2).

### 2.2. Effect of Mdivi-1 on Microglial Changes in the Substantia Nigra

Neurodegeneration in the experiment was accompanied by pronounced reactive changes in glial cells: an increase in the density of Iba1-positive microglia was observed in both halves of the substantia nigra (*p* = 0.0001) (Figure 1).

**Figure 3:**
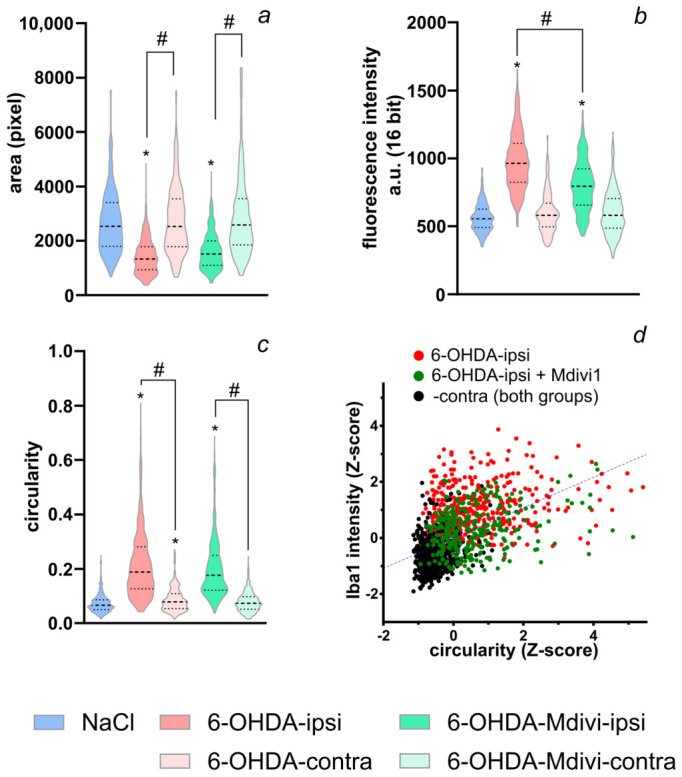
Changes in Iba1-positive microglial parameters in animals receiving 6-OHDA alone and Mdivi-1 after 6-OHDA administration. (**a**) Changes in microglial cell area (pixels); (**b**) changes in mean fluorescence intensity (a.u.) of Iba1 immunofluorescence staining; (**c**) changes in cell circularity; (**d**) correlation between circularity and Iba1 staining intensity (standardized (Z-scores)), red dots—6-OHDA alone treated group, ipsilateral; green dots—6-OHDA + Mdivi1 group ipsilateral; black dots—contralateral side in both groups and control group; blue line—linear regression trend. “*” *p* < 0.05 compared to control; “#” *p* < 0.05 for comparison indicated by bracket. Kruskal–Wallis ANOVA with post hoc Dunn’s test. Within the violin plots, the bold dashed horizontal line denotes the median; upper and lower dashed horizontal lines mark upper and lower quartiles. For group designations, see Figure 1.

The mean area of microglial cells on the toxin injection side was significantly reduced (Figure 3a) compared to the control, while the circularity was increased (Figure 3c), reflecting the emergence of amoeboid microglial forms. Conversely, opposite changes were found on the contralateral side. Administration of Mdivi-1 significantly reduced the mean fluorescence intensity of microglial cells on the toxin injection side (*p* < 0.05) (Figure 3b), which may indicate that Mdivi-1 reduced Iba1 expression. On the side contralateral to the lesion in animals receiving Mdivi-1, the circularity value did not differ from the control, in contrast to the group receiving 6-OHDA alone, in which circularity was significantly increased compared to the control in both the right and left substantia nigra. Interestingly, a positive correlation (r = 0.54; *p* < 0.001) was found between the circularity value of cells and the intensity of Iba1 immunofluorescence staining (Figure 3d).

**Figure 4:**
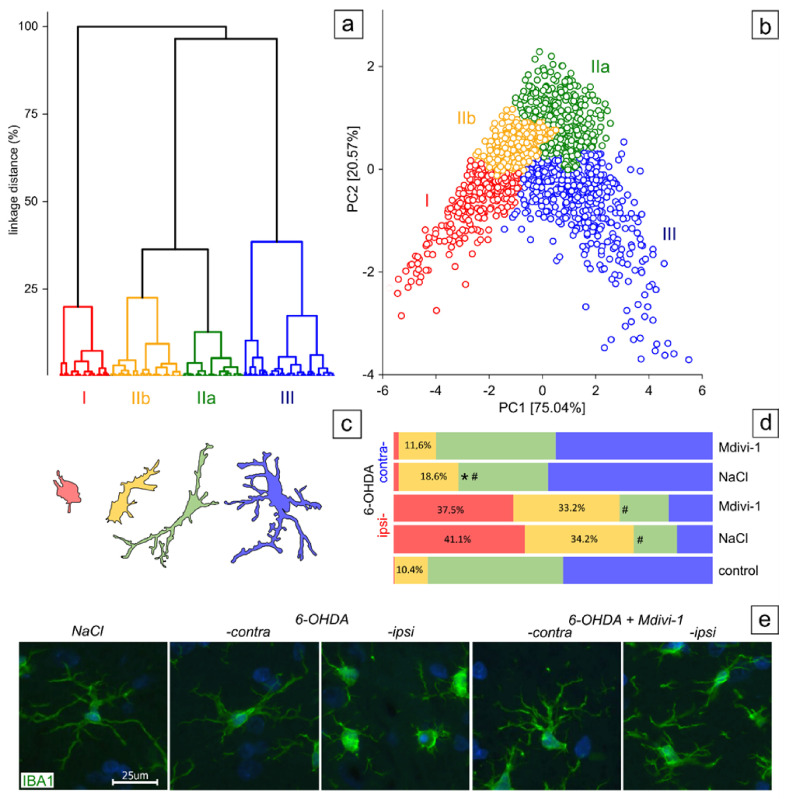
Classification of microglial cells of the substantia nigra using principal component analysis and hierarchical cluster analysis. (**a**) Clustering dendrogram (Ward’s method); distance is shown as a percentage of the maximum; Euclidean distance measure was used. (**b**) Scatter plot of data from all groups (6-OHDA, 6-OHDA + Mdivi, and control) in principal component space; colors indicate the identified clusters: red—cluster I; yellow—cluster IIb; green—cluster IIa; blue—cluster III. (**c**) Examples of outlines of representative microglial cells for each identified cluster. (**d**) Percentage of microglial cells in the substantia nigra assigned to one of four clusters in the control group following 6-OHDA administration on the ipsilateral side (ipsi-) and contralateral side (contra-). “Mdivi-1”—group receiving Mdivi-1 injections (*n* = 5); 6-OHDA—animals receiving hydroxydopamine (*n* = 5); “NaCl”—group receiving isotonic NaCl injections (*n* = 5). “*” *p* < 0.05 vs. contralateral side of Mdivi-1 group, “#” *p* < 0.05 vs control group, Chi-square test. (**e**) Immunofluorescence detection of microglia (Iba1, green; DAPI, blue), ×40 objective.

Morphological types of activated glia were identified by PCA analysis (Figure 4). 

Cluster I consisted of rounded or slightly elongated cells almost lacking processes (phagocytic Iba1-positive microglia and macrophages), in contrast to cluster III, which consisted of ramified cells characteristic of control animals. Among the intermediate clusters, more elongated cells (IIa) of ramified microglia, characteristic of the substantia nigra of control animals or transitional forms of activated microglia, were identified, while cluster IIb was represented by various types of elongated amoeboid cells or cells with short processes.

Proportion comparison for the glial cells of the identified morphological types showed that 6-OHDA administration significantly (χ2 = 344.0, *p* < 0.01) increased the proportion of activated cells (clusters I and IIb); however, on the injection side, microglial activation did not differ significantly between animals that did and did not receive Mdivi-1. In contrast, on the contralateral side, a significant reduction in the proportion of glial cells assigned to cluster IIb (χ2 = 7.869, *p* = 0.049) was found in animals receiving Mdivi-1.

Thus, we observed a trend toward a reduction in the proportion of activated microglial forms and a decrease in fluorescence intensity for Iba1 (*p* = 0.0086) on the toxin injection side under the influence of Mdivi-1. At the same time, on the contralateral side, Mdivi-1 significantly reduced the proportion of amoeboid reactive microgliocytes.

These findings suggest that Mdivi-1 administration attenuates the spread of neuroinflammatory changes and the secondary microglial response to substantia nigra destruction.

### 2.3. Effect of Mdivi-1 on Changes in Mitochondrial Proteins

**Figure 5:**
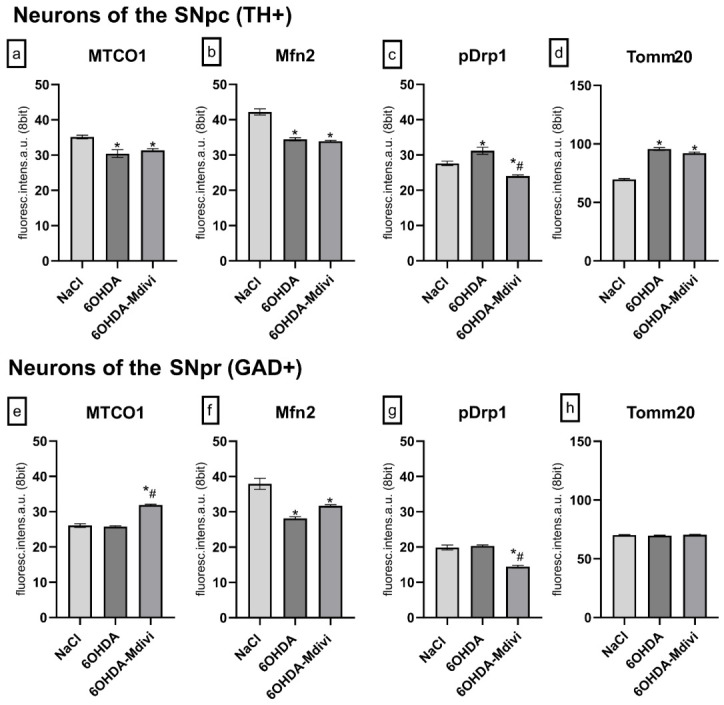
Changes in mitochondrial proteins in substantia nigra neurons. (**a**,**e**) Subunit 1 of complex IV of the respiratory chain (MTCO1); (**b**,**f**) mitochondrial fusion marker (Mfn2); (**c**,**g**) active form of the mitochondrial fission marker (pDrp1); (**d**,**h**) outer mitochondrial membrane fusion marker (Tomm20). ANOVA with post hoc Tukey test (M ± SEM). “*” *p* < 0.05 vs. control; “#” *p* < 0.05 vs. 6-OHDA. For group designations, see Figure 1.

**Figure 6:**
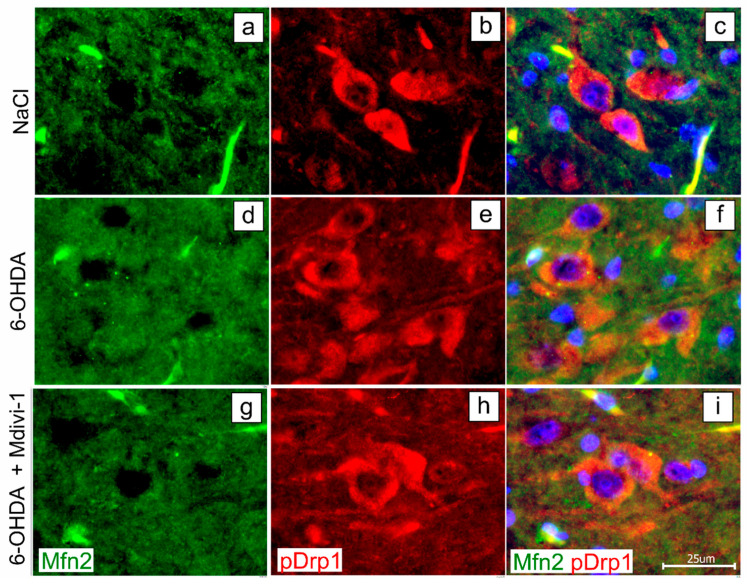
Changes in expression of mitochondrial dynamics markers. Detection of Mfn2 (**a**,**d**,**g**) and pDrp1 (**b**,**e**,**h**); (**c**,**f**,**i**) merged channels (Mfn2, green; pDrp1, red; DAPI, blue), ×40 objective. For group designations, see Figure 1.

Mitochondrial proteins, due to the almost complete destruction of dopaminergic neurons on the side of toxin administration, were assessed in cells of the contralateral substantia nigra. The data obtained demonstrated differences in the distribution of the studied markers in neurons of the SNpc and SNpr (Figure 5 and Figure 6).

Thus, in dopaminergic neurons, the expression of the outer mitochondrial membrane marker Tomm20 significantly increased after toxin administration compared to the control (*p* < 0.0001), whereas no significant changes were found in the cells of the reticular part. Treatment by Mdivi-1 did not affect the fluorescence intensity of Tomm20 in any of the studied groups.

Staining intensity for subunit 1 of complex IV of the mitochondrial respiratory chain in neurons of the SNpc was significantly reduced after toxin administration compared to the control (*p* = 0.0003) and remained at a low level after Mdivi-1 injections. In neurons of the pars reticulata, the opposite pattern was observed: in the group of animals receiving the toxin alone, immunofluorescence of this marker did not change compared to the control but increased sharply in rats after Mdivi-1 administration (*p* < 0.0001).

Parameters reflecting mitochondrial dynamics in cells of the SNpc and SNpr also exhibited divergent patterns.

Toxin administration alone led to an increase in pDrp1 staining intensity (*p* = 0.0054) and a decrease in expression of the mitochondrial fusion marker in dopaminergic neurons. Treatment by Mdivi-1 reduced pDrp1 immunofluorescence in these cells (*p* < 0.0001) and did not alter Mfn2 staining intensity.

In neurons of SNpr after the administration of the toxin was observed, a significant reduction in fluorescence of Mfn2 was observed (*p* < 0.0001), whereas pDrp1 did not differ from the control. Treatment by Mdivi-1 led to a decrease in pDrp1 content compared to animals receiving the toxin alone (*p* < 0.0001), while Mfn2 fluorescence increased and approached control values (*p* < 0.0001).

Thus, assessment of mitochondrial condition revealed changes in many mitochondrial markers in dopaminergic neurons of the contralateral SN following 6-OHDA administration. Neurons of the SNpr responded to toxin injection by decreasing the content of Mfn2. Course treatment with Mdivi-1 had virtually no effect on mitochondrial markers in dopamine neurons on the contralateral side but changed immunofluorescence intensity of mitochondrial proteins in neurons of the SNpr.

## 3. Discussion

In our experiments, mitochondrial markers were assessed in neurons of the SNpc and SNpr contralateral to the toxin injection.

Importantly, in unilateral SN damage (the most commonly used approach in toxin injection experiments), changes affect not only ipsilateral but also contralateral brain structures. Unilateral damage models of dopaminergic SN neurons may, among other things, be considered consistent with the asymmetric substantia nigra damage observed in the early stages of PD [20].

Despite the absence of direct connections between the left and right substantia nigra, mutual influences between them have been demonstrated in the literature. For example, following unilateral 6-OHDA injection, similar changes in neuronal activity in the pars reticulata were found on both the ipsilateral and contralateral sides relative to the toxin injection [25]. Similarly, synaptic changes in the contralateral striatum have been described [26]. Based on anatomical studies [27], changes in the opposite hemisphere following unilateral substantia nigra degeneration are often attributed to crossed projections of the pedunculopontine nucleus [25], which has reciprocal connections with the SNpc and SNpr and is involved in motor control [28]. Furthermore, connections of the contralateral striatum [29] and thalamus [30] have previously been shown, which may mediate the mutual influence between the damaged and intact substantia nigra, as well as support various forms of plasticity in preserved neurons under abnormal conditions. For example, Iyer et al. [31] review evidence of crossed innervation of the striatum from the substantia nigra pars compacta and suggest that a small number of neurons forming the interhemispheric nigrostriatal pathway, which crosses in the midbrain tegmentum, participate in compensating for striatal dopamine deficiency.

The increase in staining intensity of the outer mitochondrial membrane marker Tomm20 in dopaminergic neurons observed in our experiments following toxin administration, accompanied by intensified mitochondrial fission and reduced immunofluorescence of the mitochondrial fusion marker, may indicate an increase in the mitochondrial pool volume in neurons of this region of the substantia nigra. However, this does not suggest improved energy supply; rather, it supports the hypothesis of impaired clearance of damaged mitochondria. These changes in mitochondrial dynamics proteins were accompanied by decreased expression of the complex IV marker of the respiratory chain, suggesting impaired functional activity of defective organelles. Similar results have been described in many experimental models of neurodegenerative diseases [32,33].

The changes in mitochondrial markers in dopaminergic neurons on the side contralateral to the toxin injection occurred during microglial activation and were interpreted by us as secondary changes in response to the spread of the inflammation to the contralateral substantia nigra. This hypothesis is supported by the experimentally observed increase in microglial staining intensity in both parts of the substantia nigra and emergence of activated forms of microgliocytes in these regions.

Recent studies have shown that neuroinflammation is accompanied by the transition of microglia to an activated state as a result of the release of endogenous molecules associated with neural tissue damage and neuronal death (DAMPs—damage-associated molecular patterns) or exogenous pathogen-associated molecular patterns (PAMPs) [34]. In this context, microglial cells can remain in an activated state for a long time, form clusters around neurons, and cause neuronal damage and subsequent death, which triggers a “vicious cycle”: damaged and dying neurons release chemoattractants that cause an influx of activated microglia into the lesion site, which in turn causes even greater neuronal damage and death (reactive microgliosis). It is known that the release of pro-inflammatory factors and reactive oxygen species by microglia is associated with NLRP3 inflammasome signaling cascades [35] and is linked to increased mitochondrial fission in microgliocytes [36].

The above facts cast doubt on the potential of compensatory participation of dopaminergic neurons on the side contralateral to the lesion in normalizing neurotransmitter metabolism and, conversely, point to the involvement of “control” neurons in the spread of the pathological process mediated by glial cells.

In our study, course administration of Mdivi-1 did not exert a neuroprotective effect on dopaminergic neurons and did not change the mitochondrial proteins of these cells on the contralateral side: only a reduction in the mitochondrial fission marker was observed, whereas the other proteins showed no significant changes.

In neurons of the pars reticulata on the contralateral side following toxin administration, a decrease in mitochondrial fusion intensity was found, which was not accompanied by changes in other mitochondrial markers. Treatment by Mdivi-1 reduced neurodegeneration indicators in GAD-positive cells on the toxin injection side and significantly changed the immunofluorescence of mitochondrial proteins in GABA neurons on the contralateral side: with an increased fluorescence intensity of subunit 1 of complex IV, a shift in the balance of mitochondrial dynamics toward fusion of these organelles was observed, which may indirectly indicate increased bioenergetic function of mitochondria in these cells and a positive influence of Mdivi-1 on this neuronal type. Thus, in our study, we did not see the off-target effects of Mdivi-1 described in the literature: against the background of its administration, pDRP1 decreased in both groups of neurons, and neurodegeneration did not worsen. We hypothesized that Mdivi-1, in addition to a direct effect on GABAergic neurons, may also exert its influence by altering the functional state of microglial cells, the mechanisms of which require future evaluation. Dopaminergic neurons, in contrast, exhibited greater sensitivity to the effects of pro-inflammatory factors, reflected in significantly more severe changes in their mitochondrial parameters and the absence of a neuroprotective effect from course administration of the mitochondrial division inhibitor.

It has been repeatedly mentioned in recent literature that Mdivi-1 administration inhibits neuroinflammation. The data from the present study are consistent with the described reduction in microglia-induced neuroinflammation in the kainate model [37], as well as with the reduction in microglial activation and amelioration of cognitive deficits in septic patients, likely via NF-κB and Nrf2/Keap1/HO-1 signaling pathways [9]; Mdivi-1 significantly reduced the expression of pro-inflammatory markers in LPS-treated microglia [38].

## 4. Materials and Methods

### 4.1. Animal Care

Male Wistar rats (*n* = 15), 3.5 months old and weighing 300–350 g at the beginning of the experiment, were taken from the Stolbovaya Branch of the Scientific Center for Biomedical Technologies of the Federal Medical and Biological Agency. The animals were kept under conventional vivarium conditions at 20–22 °C, with a 12 h light cycle and access to water and food ad libitum. The mice were quarantined for 14 days before the start of the experiment.

Animal procedures were conducted in accordance with the Recommendation of the Eurasian Economic Commission Council No. 33 dated November 14, 2023 On Guidelines for Handling Laboratory (Experimental) Animals in Preclinical Studies, and the Guide for the Care and Use of Laboratory Animals: Eighth Edition (2011).

### 4.2. Surgical Procedures

For anesthesia during stereotaxic surgery, Zoletil 100 (Valdepharm; France; solvent, Delpharm Tours, France) at 3 mg/100 g and Xyla (Interchemie werken ‘De Adelaar’ B.V., Waalre, The Netherlands) at 3 mg/kg were administered intramuscularly to maintain anesthesia. For premedication, atropine (DALKHIMPHARM OJSC, Khabarovsk, Russia) at a dose of 0.04 mg/kg was administered subcutaneously 10–15 min before Xyla administration. Subsequently, the animals were placed in a laboratory stereotaxic frame (RWD Life Science Co., Shenzhen, LTD, China), and injections were performed using a Hamilton syringe (Hamilton Bonaduz AG, Bonaduz, Switzerland). To reproduce parkinsonian syndrome, the animals (*n* = 10) were injected with 6-OHDA hydrochloride (Sigma-Aldrich, St. Louis, MO, USA) at a dose of 12 μg in 3 μL of 0.05% ascorbic acid solution into the right pars compacta of the substantia nigra (SNpc) according to the following Paxinos atlas coordinates: AP = –4.8; L = 1.9; V = 8.0. The same volume of the carrier solution was administered in the contralateral substantia nigra. Ten days after surgery, rats (*n* = 5) received intraperitoneal injections of Mdivi-1 (Beijing Solarbio Science & Technology Co., Ltd., Beijing, China) suspension in 0.1% DMSO in isotonic NaCl solution at a dose of 20 mg/kg for 5 days. Control animals (*n* = 5) received bilateral intranigral injections of the solvent.

The initiation of Mdivi-1 injections on day 10 following 6-OHDA administration was based on the approximate timeline of microglial activation and maximal neuronal damage after lesion induction. In this experiment, Mdivi-1 administration preceded the massive loss of dopaminergic neurons in order to evaluate the agent’s effects at early, rather than advanced, stages of neurodegeneration associated with the onset of microglial involvement in the pathological process. During the planning of our experiment, we relied on the data on the dynamics of neuron death published in the literature [39]. In the study by Marinova-Mutafchieva L. et al., a significant loss of dopaminergic neurons in the substantia nigra commenced from day 9 after 6-OHDA injection, reaching 58% by day 15. Meanwhile, microglial activation began to manifest from the first day following 6-OHDA administration and also peaked by day 15. The dose was also selected based on the literature data reported in the article [40]. In our work, a medium dose was used to reduce the off-target effects and potential toxicity of the drug.

The frequency of administration was taken from consideration of the effect on the acute glial response that occurs from day 1 to 15 according to the publication [39].

### 4.3. Immunofluorescence Staining

After the completion of Mdivi-1 administration, the rats were decapitated using a guillotine. The brains were removed, fixed in 10% formalin for 24 h, then cryoprotected in sucrose, and frozen in O.C.T. compound (TissueTek O.C.T., Sakura Finetek Inc., Torrance CA, USA), and serial frontal sections (10 μm) were cut using a cryostat. For immunofluorescence staining, sections underwent heat-induced epitope retrieval using a steamer (15 min, 0.01 M Tris-EDTA buffer, pH 9.0). To phenotype neurons of the substantia nigra, antibodies against tyrosine hydroxylase (TH, 1:500, ab137869, Abcam, Cambridge, UK) and glutamate decarboxylase (GAD65/67, 1:500 ab183999, Abcam, Cambridge, UK) were used; additionally, the microglial marker protein Iba1 (1:300, ab178847, Abcam, Cambridge, UK) was detected. To assess mitochondrial changes, antibodies against subunit 1 of complex IV of the mitochondrial respiratory chain (MTCO1, 1:300, ab14705, Abcam, Cambridge, UK) and mitochondrial dynamics proteins—specifically the fission activator Drp1 phosphorylated at Ser616 (pDrp1, 1:100, K009964P, Beijing Solarbio Science & Technology Co., Ltd., Beijing, China), the mitochondrial outer membrane Tomm20 (1:150, ab56783, Abcam, Cambridge, UK), and the outer mitochondrial membrane fusion marker Mfn2 (1:200, K011410M, Solarbio)—were used. Binding was visualized using secondary antibodies (Invitrogen, Carlsbad, CA, USA) against rabbit and mouse IgG labeled with the fluorochromes Alexa 488 and Alexa 594, respectively. Negative control sections were incubated with secondary antibodies alone. For nuclear counterstaining, sections were included in Fluoroshield medium (Abcam, Cambridge, UK) containing DAPI.

### 4.4. Morphometry and Data Analysis

A Nikon Eclipse Ni-u microscope (Nikon, Japan) was used in this study. The image acquisition settings were the same for all groups. For morphometry, 4–5 serial sections per animal were analyzed using ImageJ-FIJI software, version 1.54. Measurements were performed on images of the SNpc and SNpr on the contralateral side. At least 100 neuronal cell bodies per animal were manually segmented, and nuclei were excluded from measurement. Fluorescence intensity was assessed in 8-bit grayscale units. Lesion volume was assessed by tyrosine hydroxylase staining; the SNpc in the lesioned area and on the contralateral side were outlined, and fluorescence intensity was compared. Data were expressed as a percentage for the lesioned side relative to the contralateral side and compared with controls. In morphometry, the belonging of animals to a particular group is unknown (tables of correspondence between image numbers and preparations are not available to the researcher).

To analyze changes in microglial cell morphology and to morphologically assess microglial activation, a classification of microglial cells and counting of their morphological types were performed similarly to a previously published protocol [23]. Using ImageJ software version 1.54 with the “magic wand” tool and a Wacom graphics tablet, the outline of microglial cell processes was manually traced on images obtained with a ×20 objective lens. For analysis, cells with a discernible nucleus in the region of the substantia nigra pars compacta were selected; in total, 300 cells were processed per group for both the left and right substantia nigra (from 5 sections, 60 cells per animal). The area and perimeter of the cell outline were evaluated, as well as the following shape factors: roundness, circularity, solidity, and aspect ratio (the ratio of the major to minor axis of the fitted ellipse). These parameters have been used widely in various combinations for microglial cell classification [23,41].

Statistical analysis was performed using Statistica 13.3 and GraphPad Prism 8.0.2 (GraphPad Software). For analysis of cell shape and classification, principal component analysis (PCA) and hierarchical cluster analysis (clusters were identified using Ward’s method) were used. To assess the morphology of microglial processes using PCA, based on analysis of the shape factors listed above, two components explaining more than 95% of the variance were extracted (PC1, associated with cell size and the degree of process branching; and PC2, associated with cell elongation). Subsequently, using hierarchical cluster analysis, four clusters reflecting different morphological types of activated glia were identified.

The proportions of identified cell types in the experimental groups were assessed using the chi-square test. For comparisons between groups for the parameters under study, ANOVA with the post hoc Tukey test was used. Normality of distribution was assessed using the Shapiro–Wilk test. Results are presented as mean ± SEM (standard error of the mean). Differences between groups were considered statistically significant at *p* < 0.05.

## 5. Conclusions

Unilateral administration of 6-hydroxydopamine into the substantia nigra leads not only to damage of dopaminergic neurons but also to pro-inflammatory changes in microglia and a shift in mitochondrial markers of neurons in the SNpc and SNpr on the side contralateral to the toxin injection. Treatment by Mdivi-1 did not affect the damage and mitochondrial markers of dopaminergic neurons; however, it changed immunofluorescence intensity of mitochondrial proteins in neurons of the pars reticulata on the contralateral side. Despite the positive findings, the use of Mdivi-1 to manage pathological processes in neurodegeneration requires additional studies that include a differential assessment of its effects on various cell types. The effects of Mdivi-1 must be investigated in detail on glial cells and different types of neurons following the induction of brain structure damage, which will allow determination of the real potential for its use in targeted therapy for neurodegeneration.

## Data Availability

The original contributions presented in this study are included in the article. Further inquiries can be directed to the corresponding author.
